# Crystal structure of 13-phenyl-2,3,4,13-tetra­hydro-1*H*-indazolo[1,2-*b*]phthalazine-1,6,11-trione

**DOI:** 10.1107/S2056989015023452

**Published:** 2015-12-12

**Authors:** Esma Lamera, Saida Benzerka, Abdelmalek Bouraiou, Sofiane Bouacida, Hocine Merazig, Aissa Chibani, Marc Le Borgne, Zouhair Bouaziz

**Affiliations:** a Unité de Recherche de Chimie de l’Environnement et Moléculaire Structurale CHEMS, Université des Frères Mentouri, Constantine 25000, Algeria; b Laboratoire de Synthèse des Molécules d’Intérêts Biologiques, Université des Frères Mentouri, Constantine 25000, Algeria; c Département Sciences de la Matière, Université Oum El Bouaghi, 04000, Algeria; d Université de Lyon, Université Lyon 1, Faculté de Pharmacie, ISPB, EA 4446 Biomolécules Cancer et Chimiorésistances, SFR Santé Lyon-Est CNRS UMS3453-INSERM US7, 8 Avenue Rockefeller, F-69373 Lyon Cedex 8, France

**Keywords:** crystal structure, indazolo, phthalazine-trione, C—H⋯O hydrogen bonds, π–π inter­actions

## Abstract

The title compound, C_21_H_16_N_2_O_3_, consists of an indazolone moiety, bearing a phenyl group, fused to a phthalazine ring system (r.m.s. deviation = 0.018 Å). The phenyl ring is almost normal to the mean plane of the five-membered ring of the indazolone moiety, making a dihedral angle of 89.64 (7)°. The six-membered ring of the indazolone moiety has an envelope conformation, with the central methyl­ene C atom as the flap. In the crystal, mol­ecules are linked *via* C—H⋯O hydrogen bonds, forming slabs parallel to the *bc* plane. The slabs are linked *via* C—H⋯π and π–π inter­actions [the shortest inter-centroid distance involving rings of pyrazolo­phthalazine moieties is 3.6430 (8) Å], forming a three-dimensional structure.

## Related literature   

For application of phthalazine derivatives see: Mosaddegh & Hassankhani (2011 ▸); Hasaninejed *et al.* (2012 ▸); Keshipour *et al.* (2012 ▸). For the synthesis of this class of compounds, see: Carling *et al.* (2004 ▸); Cashman & Ghirmai (2009 ▸); Hall *et al.* (1992 ▸, 2001 ▸); Bouraiou *et al.* (2015 ▸); Nomoto *et al.* (1990 ▸). For the synthesis of the title compound, see: Bouraiou *et al.* (2015 ▸); Khurana & Magoo (2009 ▸); Nagarapu *et al.* (2009 ▸).
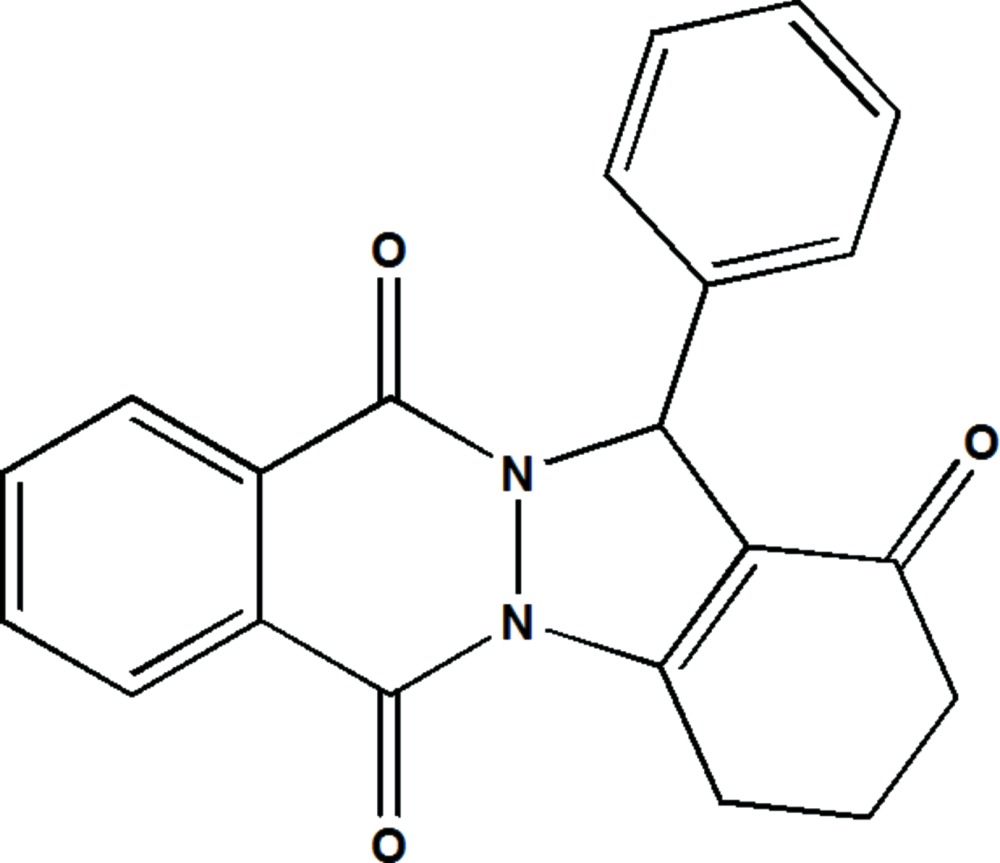


## Experimental   

### Crystal data  

C_21_H_16_N_2_O_3_
*M* *_r_* = 344.36
Monoclinic, 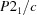

*a* = 8.9028 (2) Å
*b* = 11.4507 (3) Å
*c* = 17.0274 (4) Å
β = 104.618 (1)°
*V* = 1679.64 (7) Å^3^
*Z* = 4
Mo *K*α radiation
μ = 0.09 mm^−1^
*T* = 295 K
0.13 × 0.09 × 0.05 mm

### Data collection  

Bruker APEXII diffractometer
Absorption correction: multi-scan (*SADABS*; Bruker, 2011 ▸) *T*_min_ = 0.957, *T*_max_ = 0.986
4873 measured reflections
4873 independent reflections
3617 reflections with *I* > 2σ(*I*)

### Refinement  

*R*[*F* ^2^ > 2σ(*F* ^2^)] = 0.054
*wR*(*F* ^2^) = 0.182
*S* = 1.12
4873 reflections
235 parameters
H-atom parameters constrained
Δρ_max_ = 0.6 e Å^−3^
Δρ_min_ = −0.28 e Å^−3^

### 

Data collection: *APEX2* (Bruker, 2011 ▸); cell refinement: *SAINT* (Bruker, 2011 ▸); data reduction: *SAINT*; program(s) used to solve structure: *SIR2002* (Burla *et al.*, 2005 ▸); program(s) used to refine structure: *SHELXL97* (Sheldrick, 2008 ▸); molecular graphics: *ORTEP-3 for Windows* (Farrugia, 2012 ▸); software used to prepare material for publication: *WinGX* (Farrugia, 2012 ▸).

## Supplementary Material

Crystal structure: contains datablock(s) I. DOI: 10.1107/S2056989015023452/su5255sup1.cif

Structure factors: contains datablock(s) I. DOI: 10.1107/S2056989015023452/su5255Isup2.hkl

Supporting information file. DOI: 10.1107/S2056989015023452/su5255Isup3.cml

**Fig. 1.**
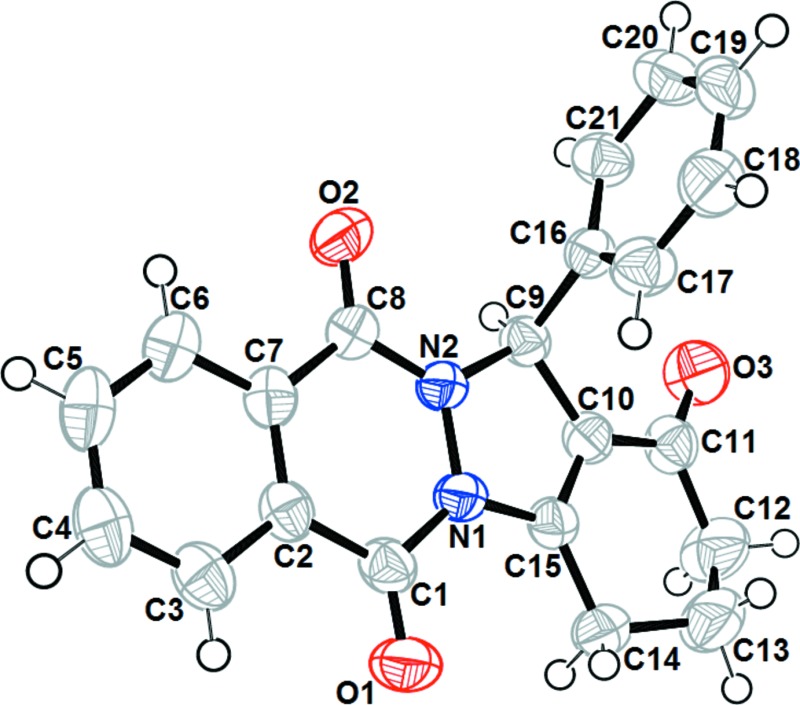
The molecule structure of the title compound, with atom labelling. Displacement are drawn at the 50% probability level.

**Fig. 2.**
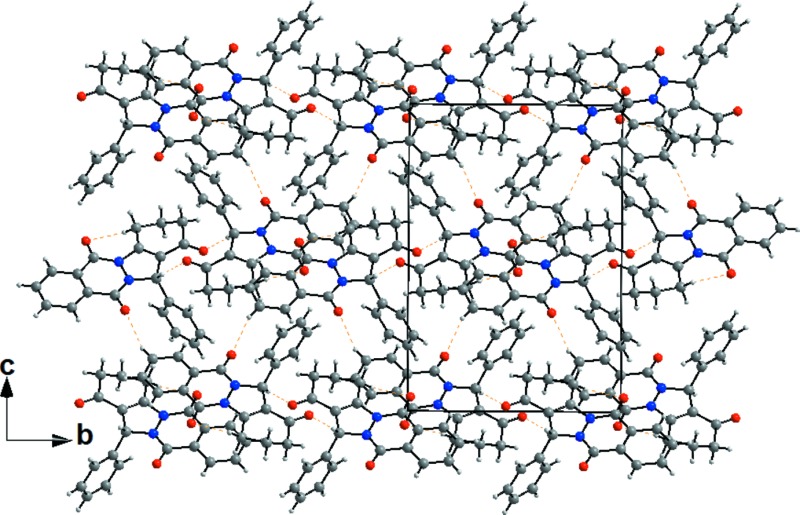
A view along the a axis of the crystal packing of the title compound, showing the C—H···O hydrogen bonds as dashed lines (see Table 1).

. DOI: 10.1107/S2056989015023452/su5255fig1.tif
The mol­ecule structure of the title compound, with atom labelling. Displacement are drawn at the 50% probability level.

a . DOI: 10.1107/S2056989015023452/su5255fig2.tif
A view along the *a* axis of the crystal packing of the title compound, showing the C—H⋯O hydrogen bonds as dashed lines (see Table 1).

CCDC reference: 1440831

Additional supporting information:  crystallographic information; 3D view; checkCIF report

## Figures and Tables

**Table 1:** Hydrogen-bond geometry (Å, °) *Cg*3 is the centroid of the C2–C7 ring.

*D*—H⋯*A*	*D*—H	H⋯*A*	*D*⋯*A*	*D*—H⋯*A*
C5—H5⋯O2^i^	0.93	2.59	3.488 (2)	163
C9—H9⋯O3^ii^	0.98	2.54	3.501 (2)	165
C18—H18⋯*Cg*3^iii^	0.93	2.68	3.552 (2)	156

Symmetry codes: (i) 
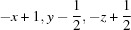
; (ii) 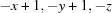; (iii) 
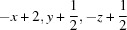
.

## supplementary crystallographic information

### S1. Comments

Phthalazine derivatives have been reported to act as anti­convulsants (Carling *et al.* 2004), potential inhibitors of serotonin reuptake (Cashman & Ghirmai, 2009), anti-proliferative agents against different human and murine tumor cells (Hall *et al.*, 1992, 2001) and vasorelaxant agents (Nomoto *et al.* 1990). Therefore, a number of synthetic methods have been developed in recent years to uncover a variety of new reagents for the synthesis of phthalazine derivatives (Mosaddegh & Hassankhani, 2011; Hasaninejed *et al.*, 2012; Keshipour *et al.*, 2012). In previous work, we have reported the synthesis and structure heterocyclic compounds bearing a phthalazine unit (Bouraiou *et al.*, 2015). Herein, we describe the synthesis and crystal structure of the title indazolo phthalazine-trione derivative, resulting from the reaction of phthalhydrazide, cyclo­hexa-1,3-dione and benzaldehyde in the presence of catalytic amounts of sulfuric acid.

The title compound, Fig. 1, consists of an indazolone moiety, bearing a phenyl group, fused to a phthalazine ring system [r.m.s. deviation = 0.018 Å]. The phenyl ring (C16—C21) is almost normal to the mean plane of the five-membered ring (N1/N2/C9/C10/C15) of the indazolone moiety with a dihedral angle of 89.64 (7)°. The six-membered ring of the indazolone moiety (C10—C15) has an envelope conformation with the central methyl­ene C atom, C13, as the flap.

In the crystal, molecules are linked *via* C—H···O hydrogen bonds forming slabs parallel to the *bc* plane (Table 1 and Fig. 2). The slabs are linked *via* C—H···π (Table 1) and slipped parallel π-π inter­actions [the shortest inter-centroid distance involves *Cg*2···*Cg*3^i^ = 3.6430 (8) Å; *Cg*2 and *Cg*3 are the centroids of rings (N1/N2/C1/C2/C7/C8) and (C2—C7), respectively; inter-planar distance = 3.457 (5); slippage = 1.07 Å; symmetry code: (i) −*x* + 1, −*y*, −*z*], forming a three-dimensional structure.

### S2. Synthesis and crystallization

The title compound was synthesized in accordance with established methods (Khurana & Magoo, 2009; Bouraiou *et al.*, 2015). Spectroscopic results and physical properties are in agreement with literature reports (Nagarapu *et al.*, 2009). The solid obtained, was recrystallized in a hot CHCl_3_/EtOAc/EtOH (1:1:1) mixture giving yellow crystals of the title compound. MS (ES-API): m/*z* [*M*+H]^+^ = 345.1; ^1^H-NMR δ (p.p.m.) (250 MHz, CDCl_3_): 8.35–8.23 (m, 2H), 7.88–7.84 (m, 2H), 7.32–7.30 (m, 5H), 6.46 (s, 1H), 3.63–3.30 (m, 2H), 2.51–2.46 (m, 2H), 2.32–2.22 (m, 2H); ^13^C-NMR δ (p.p.m.) (62.9 MHz, _3_): 192.6, 156.1, 154.3, 152.3, 136.4, 134.6, 133.6, 129.1, 129.0, 128.7, 128.0, 127.8, 127.2, 119.7, 65.0, 37.0, 24.6, 22.3.

### S3. Refinement

Crystal data, data collection and structure refinement details are summarized in Table 2. The H atoms were located in difference Fourier maps but introduced in calculated positions and refined as riding atoms: C—H = 0.93–0.98 Å with *U*_iso_(H) = 1.2 *U*_eq_(C).

### Crystal data

**Table:**

C_21_H_16_N_2_O_3_	*F*(000) = 720
*M**_r_* = 344.36	*D*_x_ = 1.362 Mg m^−^^3^
Monoclinic, *P*2_1_/*c*	Mo *K*α radiation, λ = 0.71073 Å
Hall symbol: -P 2ybc	Cell parameters from 4002 reflections
*a* = 8.9028 (2) Å	θ = 3.0–30.5°
*b* = 11.4507 (3) Å	µ = 0.09 mm^−^^1^
*c* = 17.0274 (4) Å	*T* = 295 K
β = 104.618 (1)°	Prism, yellow
*V* = 1679.64 (7) Å^3^	0.13 × 0.09 × 0.05 mm
*Z* = 4	

### Data collection

**Table:**

Bruker APEXII diffractometer	4873 independent reflections
Radiation source: Enraf Nonius FR590	3617 reflections with *I* > 2σ(*I*)
Graphite monochromator	*R*_int_ = 0
CCD rotation images, thick slices scans	θ_max_ = 30.6°, θ_min_ = 3.4°
Absorption correction: multi-scan (*SADABS*; Bruker, 2011)	*h* = −12→12
*T*_min_ = 0.957, *T*_max_ = 0.986	*k* = 0→16
4873 measured reflections	*l* = 0→23

### Refinement

**Table:**

Refinement on *F*^2^	Primary atom site location: structure-invariant direct methods
Least-squares matrix: full	Secondary atom site location: difference Fourier map
*R*[*F*^2^ > 2σ(*F*^2^)] = 0.054	Hydrogen site location: inferred from neighbouring sites
*wR*(*F*^2^) = 0.182	H-atom parameters constrained
*S* = 1.12	*w* = 1/[σ^2^(*F*_o_^2^) + (0.1115*P*)^2^ + 0.0058*P*] where *P* = (*F*_o_^2^ + 2*F*_c_^2^)/3
4873 reflections	(Δ/σ)_max_ < 0.001
235 parameters	Δρ_max_ = 0.6 e Å^−^^3^
0 restraints	Δρ_min_ = −0.28 e Å^−^^3^

### Special details

**Table:**

Geometry. All e.s.d.'s (except the e.s.d. in the dihedral angle between two l.s. planes) are estimated using the full covariance matrix. The cell e.s.d.'s are taken into account individually in the estimation of e.s.d.'s in distances, angles and torsion angles; correlations between e.s.d.'s in cell parameters are only used when they are defined by crystal symmetry. An approximate (isotropic) treatment of cell e.s.d.'s is used for estimating e.s.d.'s involving l.s. planes.
Refinement. Refinement of *F*^2^ against ALL reflections. The weighted *R*-factor *wR* and goodness of fit *S* are based on *F*^2^, conventional *R*-factors *R* are based on *F*, with *F* set to zero for negative *F*^2^. The threshold expression of *F*^2^ > σ(*F*^2^) is used only for calculating *R*-factors(gt) *etc*. and is not relevant to the choice of reflections for refinement. *R*-factors based on *F*^2^ are statistically about twice as large as those based on *F*, and *R*- factors based on ALL data will be even larger.

### Fractional atomic coordinates and isotropic or equivalent isotropic displacement parameters (Å^2^)

**Table:**

	*x*	*y*	*z*	*U*_iso_*/*U*_eq_	
C1	0.75069 (15)	0.02395 (11)	0.00764 (7)	0.0373 (3)	
C2	0.68676 (14)	−0.05024 (11)	0.06179 (7)	0.0350 (3)	
C3	0.69309 (16)	−0.17117 (12)	0.05347 (9)	0.0452 (3)	
H3	0.7353	−0.2031	0.0136	0.054*	
C4	0.63708 (18)	−0.24340 (13)	0.10405 (10)	0.0519 (4)	
H4	0.6422	−0.324	0.0986	0.062*	
C5	0.57284 (17)	−0.19596 (15)	0.16328 (10)	0.0525 (4)	
H5	0.5359	−0.245	0.1977	0.063*	
C6	0.56360 (16)	−0.07659 (14)	0.17128 (9)	0.0455 (3)	
H6	0.5189	−0.0454	0.2105	0.055*	
C7	0.62114 (13)	−0.00234 (12)	0.12069 (7)	0.0359 (3)	
C8	0.60888 (14)	0.12553 (12)	0.12975 (7)	0.0369 (3)	
C9	0.66558 (13)	0.31891 (11)	0.07410 (7)	0.0332 (3)	
H9	0.5574	0.3454	0.0611	0.04*	
C10	0.73305 (14)	0.33728 (11)	0.00262 (7)	0.0350 (3)	
C11	0.74291 (16)	0.44774 (12)	−0.03792 (8)	0.0425 (3)	
C12	0.8090 (2)	0.43883 (17)	−0.11124 (11)	0.0664 (5)	
H12A	0.7242	0.4281	−0.1592	0.08*	
H12B	0.8599	0.5119	−0.1175	0.08*	
C13	0.9230 (3)	0.34114 (17)	−0.10670 (12)	0.0689 (5)	
H13A	1.0152	0.358	−0.0639	0.083*	
H13B	0.9534	0.3375	−0.1575	0.083*	
C14	0.85967 (17)	0.22277 (14)	−0.09042 (8)	0.0464 (3)	
H14A	0.7873	0.1942	−0.1392	0.056*	
H14B	0.9439	0.167	−0.0747	0.056*	
C15	0.77933 (14)	0.23569 (11)	−0.02380 (7)	0.0347 (3)	
C16	0.75871 (13)	0.37878 (11)	0.15078 (7)	0.0329 (3)	
C17	0.91360 (15)	0.35115 (14)	0.18289 (8)	0.0455 (3)	
H17	0.9599	0.2926	0.1593	0.055*	
C18	1.00001 (17)	0.41101 (16)	0.25044 (8)	0.0532 (4)	
H18	1.1036	0.3916	0.2723	0.064*	
C19	0.93283 (19)	0.49868 (14)	0.28486 (8)	0.0504 (4)	
H19	0.9917	0.5398	0.3291	0.06*	
C20	0.7781 (2)	0.52582 (13)	0.25386 (8)	0.0510 (4)	
H20	0.7319	0.5841	0.2778	0.061*	
C21	0.69169 (16)	0.46548 (12)	0.18660 (8)	0.0414 (3)	
H21	0.5875	0.4839	0.1656	0.05*	
N1	0.74174 (12)	0.14291 (9)	0.02082 (6)	0.0351 (2)	
N2	0.67235 (12)	0.19043 (10)	0.07981 (6)	0.0355 (2)	
O1	0.80699 (15)	−0.01280 (10)	−0.04590 (6)	0.0574 (3)	
O2	0.54814 (13)	0.17285 (10)	0.17821 (6)	0.0539 (3)	
O3	0.69339 (14)	0.53847 (10)	−0.01668 (7)	0.0591 (3)	

### Atomic displacement parameters (Å^2^)

**Table:**

	*U*^11^	*U*^22^	*U*^33^	*U*^12^	*U*^13^	*U*^23^
C1	0.0465 (6)	0.0302 (6)	0.0353 (6)	0.0009 (5)	0.0105 (5)	0.0003 (5)
C2	0.0378 (6)	0.0291 (6)	0.0350 (6)	−0.0002 (5)	0.0031 (4)	0.0034 (4)
C3	0.0509 (7)	0.0333 (7)	0.0476 (7)	0.0004 (6)	0.0055 (6)	0.0010 (5)
C4	0.0562 (8)	0.0319 (7)	0.0630 (9)	−0.0035 (6)	0.0064 (7)	0.0072 (6)
C5	0.0506 (7)	0.0452 (8)	0.0599 (9)	−0.0093 (7)	0.0109 (6)	0.0166 (7)
C6	0.0442 (7)	0.0452 (8)	0.0483 (7)	−0.0045 (6)	0.0139 (5)	0.0077 (6)
C7	0.0345 (6)	0.0353 (7)	0.0359 (6)	−0.0030 (5)	0.0049 (4)	0.0035 (5)
C8	0.0389 (6)	0.0371 (7)	0.0362 (6)	−0.0031 (5)	0.0120 (4)	0.0007 (5)
C9	0.0361 (5)	0.0289 (6)	0.0347 (6)	0.0007 (5)	0.0093 (4)	−0.0005 (4)
C10	0.0397 (6)	0.0333 (7)	0.0314 (5)	−0.0019 (5)	0.0079 (4)	0.0008 (4)
C11	0.0483 (7)	0.0347 (7)	0.0437 (7)	−0.0015 (6)	0.0103 (5)	0.0054 (5)
C12	0.0946 (13)	0.0509 (10)	0.0660 (10)	0.0039 (9)	0.0433 (9)	0.0181 (8)
C13	0.0916 (13)	0.0537 (11)	0.0784 (11)	−0.0096 (9)	0.0530 (10)	0.0009 (9)
C14	0.0575 (8)	0.0435 (8)	0.0449 (7)	−0.0030 (7)	0.0254 (6)	−0.0026 (6)
C15	0.0395 (6)	0.0325 (6)	0.0325 (6)	−0.0035 (5)	0.0097 (4)	−0.0003 (5)
C16	0.0388 (6)	0.0304 (6)	0.0307 (5)	−0.0023 (5)	0.0110 (4)	0.0020 (4)
C17	0.0405 (6)	0.0532 (9)	0.0426 (6)	0.0032 (6)	0.0101 (5)	−0.0055 (6)
C18	0.0423 (7)	0.0717 (12)	0.0419 (7)	−0.0076 (7)	0.0037 (5)	0.0002 (7)
C19	0.0636 (9)	0.0497 (8)	0.0348 (6)	−0.0164 (7)	0.0068 (6)	−0.0031 (6)
C20	0.0734 (10)	0.0380 (7)	0.0417 (7)	0.0005 (7)	0.0145 (6)	−0.0052 (6)
C21	0.0491 (7)	0.0354 (7)	0.0399 (6)	0.0057 (6)	0.0115 (5)	0.0002 (5)
N1	0.0445 (5)	0.0300 (5)	0.0341 (5)	−0.0001 (4)	0.0159 (4)	−0.0015 (4)
N2	0.0449 (5)	0.0295 (5)	0.0362 (5)	−0.0021 (4)	0.0178 (4)	−0.0020 (4)
O1	0.0895 (8)	0.0395 (6)	0.0544 (6)	0.0026 (6)	0.0389 (6)	−0.0047 (5)
O2	0.0670 (7)	0.0471 (7)	0.0603 (6)	−0.0031 (5)	0.0394 (5)	−0.0030 (5)
O3	0.0773 (8)	0.0356 (6)	0.0685 (7)	0.0087 (5)	0.0262 (6)	0.0081 (5)

### Geometric parameters (Å, º)

**Table:**

C1—O1	1.2206 (16)	C11—C12	1.512 (2)
C1—N1	1.3861 (16)	C12—C13	1.499 (3)
C1—C2	1.4700 (18)	C12—H12A	0.97
C2—C7	1.3942 (19)	C12—H12B	0.97
C2—C3	1.3945 (19)	C13—C14	1.520 (2)
C3—C4	1.375 (2)	C13—H13A	0.97
C3—H3	0.93	C13—H13B	0.97
C4—C5	1.389 (3)	C14—C15	1.4932 (19)
C4—H4	0.93	C14—H14A	0.97
C5—C6	1.378 (2)	C14—H14B	0.97
C5—H5	0.93	C15—N1	1.3951 (16)
C6—C7	1.3964 (18)	C16—C21	1.3775 (18)
C6—H6	0.93	C16—C17	1.3857 (18)
C7—C8	1.4793 (19)	C17—C18	1.3921 (19)
C8—O2	1.2215 (16)	C17—H17	0.93
C8—N2	1.3554 (16)	C18—C19	1.373 (2)
C9—N2	1.4746 (17)	C18—H18	0.93
C9—C10	1.5019 (17)	C19—C20	1.381 (2)
C9—C16	1.5217 (16)	C19—H19	0.93
C9—H9	0.98	C20—C21	1.3915 (19)
C10—C15	1.3487 (18)	C20—H20	0.93
C10—C11	1.4541 (18)	C21—H21	0.93
C11—O3	1.2187 (18)	N1—N2	1.4142 (14)
			
O1—C1—N1	120.73 (12)	H12A—C12—H12B	107.6
O1—C1—C2	124.48 (13)	C12—C13—C14	113.43 (16)
N1—C1—C2	114.78 (11)	C12—C13—H13A	108.9
C7—C2—C3	119.92 (13)	C14—C13—H13A	108.9
C7—C2—C1	121.51 (12)	C12—C13—H13B	108.9
C3—C2—C1	118.57 (13)	C14—C13—H13B	108.9
C4—C3—C2	120.21 (15)	H13A—C13—H13B	107.7
C4—C3—H3	119.9	C15—C14—C13	108.72 (12)
C2—C3—H3	119.9	C15—C14—H14A	109.9
C3—C4—C5	120.01 (15)	C13—C14—H14A	109.9
C3—C4—H4	120	C15—C14—H14B	109.9
C5—C4—H4	120	C13—C14—H14B	109.9
C6—C5—C4	120.36 (14)	H14A—C14—H14B	108.3
C6—C5—H5	119.8	C10—C15—N1	109.86 (11)
C4—C5—H5	119.8	C10—C15—C14	125.73 (12)
C5—C6—C7	120.17 (15)	N1—C15—C14	124.41 (12)
C5—C6—H6	119.9	C21—C16—C17	119.28 (12)
C7—C6—H6	119.9	C21—C16—C9	120.10 (11)
C2—C7—C6	119.32 (13)	C17—C16—C9	120.54 (11)
C2—C7—C8	121.32 (11)	C16—C17—C18	120.07 (14)
C6—C7—C8	119.35 (12)	C16—C17—H17	120
O2—C8—N2	120.42 (13)	C18—C17—H17	120
O2—C8—C7	124.50 (12)	C19—C18—C17	120.21 (13)
N2—C8—C7	115.08 (11)	C19—C18—H18	119.9
N2—C9—C10	100.09 (10)	C17—C18—H18	119.9
N2—C9—C16	112.86 (9)	C18—C19—C20	120.09 (13)
C10—C9—C16	112.92 (10)	C18—C19—H19	120
N2—C9—H9	110.2	C20—C19—H19	120
C10—C9—H9	110.2	C19—C20—C21	119.60 (14)
C16—C9—H9	110.2	C19—C20—H20	120.2
C15—C10—C11	122.13 (12)	C21—C20—H20	120.2
C15—C10—C9	111.58 (11)	C16—C21—C20	120.74 (13)
C11—C10—C9	126.22 (12)	C16—C21—H21	119.6
O3—C11—C10	122.07 (13)	C20—C21—H21	119.6
O3—C11—C12	123.22 (13)	C1—N1—C15	128.95 (11)
C10—C11—C12	114.58 (13)	C1—N1—N2	123.12 (11)
C13—C12—C11	114.02 (14)	C15—N1—N2	107.56 (10)
C13—C12—H12A	108.7	C8—N2—N1	124.10 (11)
C11—C12—H12A	108.7	C8—N2—C9	124.87 (11)
C13—C12—H12B	108.7	N1—N2—C9	110.79 (9)
C11—C12—H12B	108.7		

### Hydrogen-bond geometry (Å, º)

Cg3 is the centroid of the C2–C7 ring.

**Table:**

*D*—H···*A*	*D*—H	H···*A*	*D*···*A*	*D*—H···*A*
C5—H5···O2^i^	0.93	2.59	3.488 (2)	163
C9—H9···O3^ii^	0.98	2.54	3.501 (2)	165
C18—H18···*Cg*3^iii^	0.93	2.68	3.552 (2)	156

Symmetry codes: (i) −*x*+1, *y*−1/2, −*z*+1/2; (ii) −*x*+1, −*y*+1, −*z*; (iii) −*x*+2, *y*+1/2, −*z*+1/2.

## References

[bb1] Bouraiou, A., Bouacida, S., Merazig, H., Chibani, A. & Bouaziz, Z. (2015). *Acta Cryst.* E**71**, o604–o605. 10.1107/S2056989015013894 PMC4571420 26396820

[bb2] Bruker (2011). *APEX2*, *SAINT* and *SADABS*. Bruker AXS Inc., Madison, Wisconsin, USA.

[bb3] Burla, M. C., Caliandro, R., Camalli, M., Carrozzini, B., Cascarano, G. L., De Caro, L., Giacovazzo, C., Polidori, G. & Spagna, R. (2005). *J. Appl. Cryst.* **38**, 381–388.

[bb4] Carling, R. W., Moore, K. W., Street, L. J., Wild, D., Isted, C., Leeson, P. D., Thomas, S., O’Connor, D., McKernan, R. M., Quirk, K., Cook, S. M., Atack, J. R., Wafford, K. A., Thompson, S. A., Dawson, G. R., Ferris, P. & Castro, J. L. (2004). *J. Med. Chem.* **47**, 1807–1822. 10.1021/jm031020p 15027873

[bb5] Cashman, J. R. & Ghirmai, S. (2009). *Bioorg. Med. Chem.* **17**, 6890–6897. 10.1016/j.bmc.2009.08.025 19740668

[bb6] Farrugia, L. J. (2012). *J. Appl. Cryst.* **45**, 849–854.

[bb7] Hall, I. H., Covington, D. W., Wheaton, J. R., Izydore, R. A. & Zhou, X. (2001). *Pharmazie*, **56**, 168–174. 11234348

[bb8] Hall, I. H., Hall, E. S. & Wong, O. T. (1992). *Anticancer Drugs*, **3**, 55–62. 10.1097/00001813-199202000-00010 1623217

[bb9] Hasaninejed, A., Kazerooni, M. R. & Zare, A. (2012). *Catal. Today*, **196**, 148–155.

[bb10] Keshipour, S., Shojaei, S. & Shaabani, A. (2012). *Tetrahedron*, **68**, 6141–6145.

[bb11] Khurana, J. M. & Magoo, D. (2009). *Tetrahedron Lett.* **50**, 7300–7303.

[bb12] Mosaddegh, E. & Hassankhani, A. (2011). *Tetrahedron Lett.* **52**, 488–490.

[bb13] Nagarapu, L., Bantu, R. & Mereyala, H. B. (2009). *J. Heterocycl. Chem.* **46**, 728–731.

[bb14] Nomoto, Y., Obase, H., Takai, H., Teranishi, M., Nakamura, J. & Kubo, K. (1990). *Chem. Pharm. Bull.* **38**, 2179–2183. 10.1248/cpb.38.2179 2279280

[bb15] Sheldrick, G. M. (2008). *Acta Cryst.* A**64**, 112–122. 10.1107/S0108767307043930 18156677

