# Supplementary figures and images for: Crystal structure of 13-phenyl-2,3,4,13-tetra­hydro-1H-indazolo[1,2-b]phthalazine-1,6,11-trione
Source: Acta Crystallogr E Crystallogr Commun. 2015 Dec 12;71(Pt 12):o1036–7. doi: 10.1107/S2056989015023452 (PMC4719966; doi:10.1107/S2056989015023452)

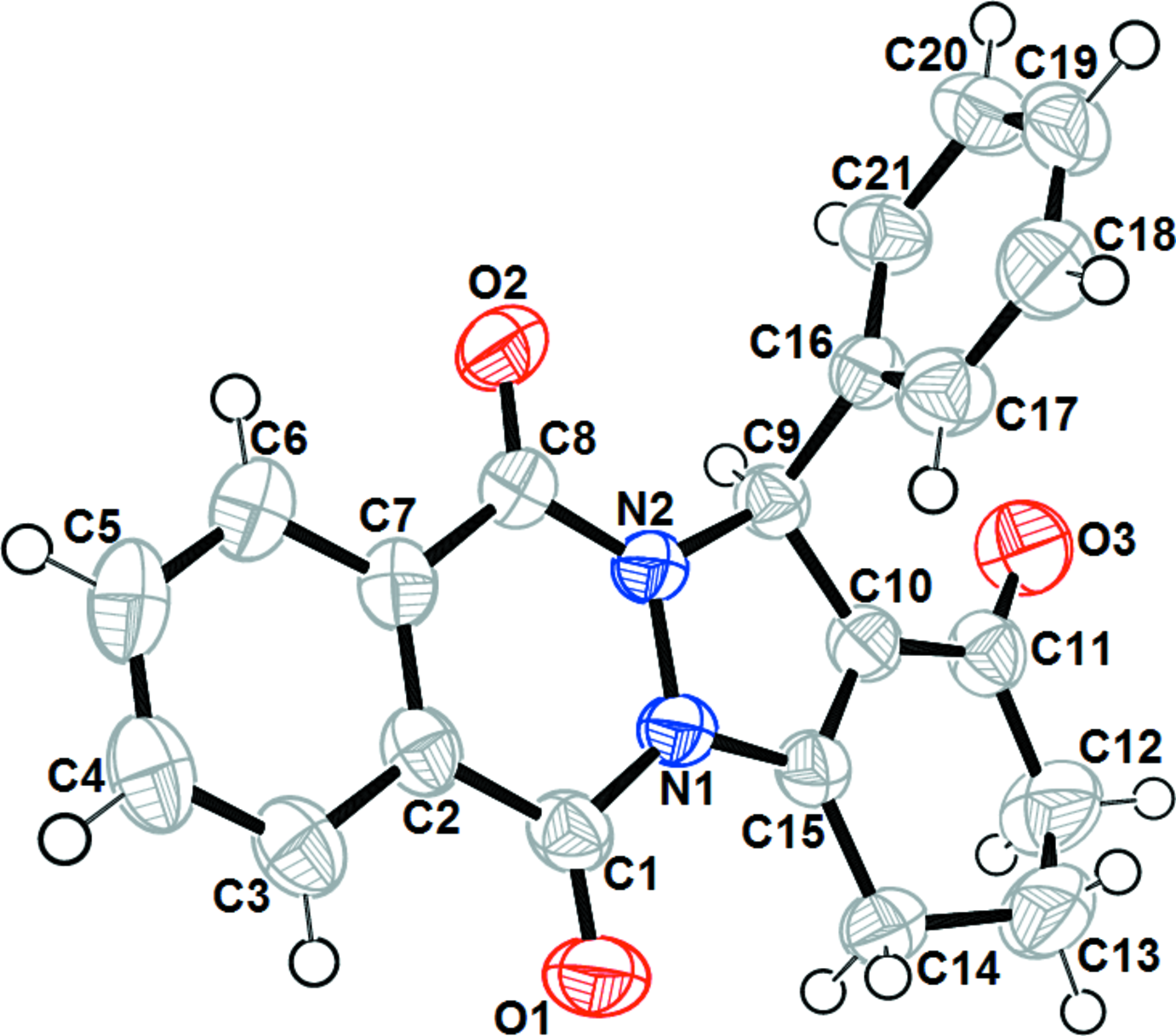

Supplement: Supplementary file 4 [file e-71-o1036-fig1.tif]

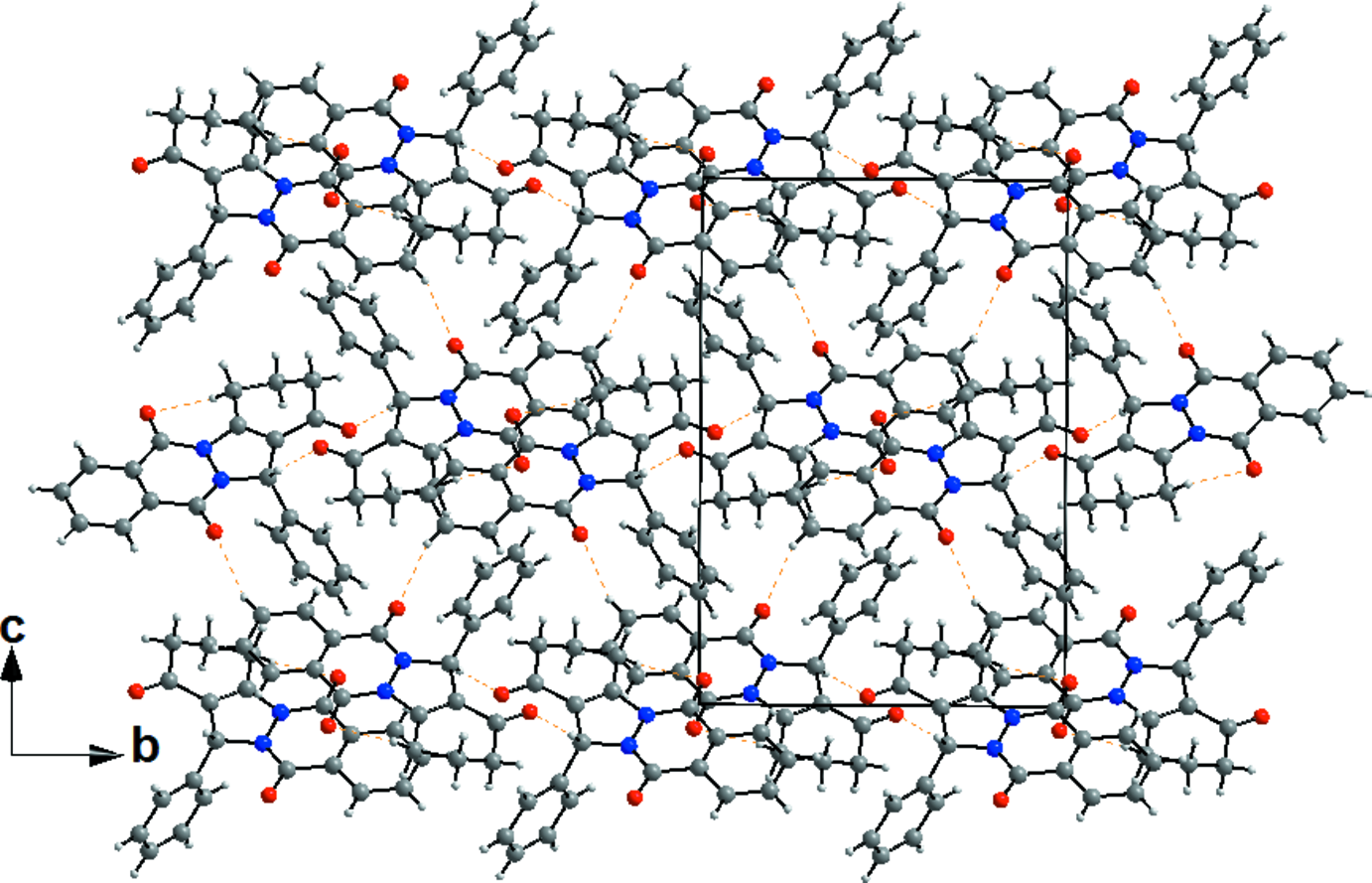

Supplement: Supplementary file 5 [file e-71-o1036-fig2.tif]
